# Association of Eosinophil Counts and Ratios With Clinical Outcomes After Acute Ischemic Stroke

**DOI:** 10.1002/brb3.71320

**Published:** 2026-03-31

**Authors:** Yangying Lu, Lei Zhang, Hongyi Yan, Yuesong Pan, Jing Jing, Xia Meng, Xingquan Zhao, Liping Liu, Yilong Wang, Yongjun Wang, Honglian Wang, Wenchao Li, Bihong Zhu

**Affiliations:** ^1^ Department of Neurology Huangyan Hospital of Wenzhou Medical University Taizhou China; ^2^ Department of Neurology Taizhou First People's Hospital Zhejiang China; ^3^ Department of Neurology Beijing Changping District Hospital Beijing China; ^4^ Department of Neurology, Beijing Tiantan Hospital Capital Medical University Beijing China; ^5^ China National Clinical Research Center For Neurological Diseases Beijing China; ^6^ Beijing Changping Hospital of Traditional Chinese Medicine Beijing China

## Abstract

**Background:**

Evidence links eosinopenia to poor 3‐month outcomes after stroke, but its long‐term prognostic value in acute ischemic cerebrovascular events remains unclear. This study aimed to evaluate the association between eosinophil counts (and ratios) and 5‐year clinical outcomes in patients with acute ischemic stroke or transient ischemic attack (TIA).

**Methods:**

We analyzed data from the Third China National Stroke Registry (CNSR‐III). Patients with acute ischemic stroke or TIA were categorized into quartiles based on eosinophil counts and ratios measured within 24 h of admission. Using the highest quartile as the reference, we calculated hazard ratios (HRs) or odds ratios (ORs) with 95% confidence intervals (CIs) for adverse outcomes. The association of eosinophil levels with 5‐year risks of stroke recurrence, poor functional outcome, all‐cause death, and composite vascular events was evaluated.

**Results:**

A total of 12,745 participants were enrolled. Compared to the fourth quartile of eosinophil counts, the first quartile were associated with an increased risk of stroke recurrence (adjusted hazard ratio [HR] = 1.25, 95% confidence interval [CI]: 1.10–1.41) and ischemic stroke recurrence (adjusted HR, 1.25; 95% CI: 1.10–1.42). Similar associations were observed over 5 years for poor functional outcome (adjusted odds ratio [OR] = 1.57, 95% CI: 1.40–1.76), composite vascular events (adjusted HR = 1.15, 95% CI: 1.02–1.29), and all‐cause death (adjusted HR = 1.53, 95% CI: 1.32–1.77). Parallel results were found for eosinophil ratios.

**Conclusions:**

This study demonstrated that low levels of both eosinophil counts and eosinophil ratios were associated with an increased risk of adverse clinical outcomes at 5 years of follow‐up in patients with ischemic stroke or TIA.

## Graphical abstract

This study demonstrates for the first time that low peripheral eosinophil levels are independent predictors of adverse 5‐year outcomes in ischemic stroke or TIA patients, providing a novel basis for risk stratification and future mechanistic research.

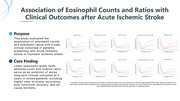

## 1 Background

Stroke is defined as a sudden onset of focal neurological dysfunction caused by an acute disturbance of the cerebral blood supply, resulting in infarction or hemorrhage of brain tissue, and lasting more than 24 h or leading to death, with ischemic stroke accounting for approximately 87% of all cases and thus being the most common etiology (Kleindorfer et al. 2021). Ischemic stroke also has high incidence, mortality, and disability rates, placing a substantial medical and economic burden on society (Herpich and Rincon 2020).

Acute ischemic stroke (AIS) not only is a vascular disease, but also is an immune‐mediated condition. Inflammation plays an important role in the pathogenesis and prognosis of this disease (Jurcau and Simion 2021). It has increasingly been demonstrated that inflammatory responses are associated with all stages of ischemic stroke, affecting both acute and chronic phases of the disease and even the clinical prognosis (Jurcau and Simion 2021; DiSabato et al. 2016; Schaeffer and Iadecola 2021; Jung et al. 2013). After an AIS, various circulating inflammatory cytokines, like all kinds of leukocyte subtypes, infiltrate the brain parenchyma (Jin et al. 2013). Prior studies have showed that circulating inflammatory factors such as IL‐6 and IL‐37 were associated with the adverse functional outcomes of ischemic stroke (Coveney et al. 2022; Holmes et al. 2017). In addition to cytokines, other inflammatory markers, such as neutrophil, lymphocyte, and monocyte counts, can also infiltrate the ischemic brain (Jurcau and Simion 2021). Eosinophils, as one of the circulating biomarkers of inflammation, has raised widespread concern. Eosinophils can regulate the immune responses by facilitating the resolution of inflammation, which assumes a pivotal role in AIS (Iadecola et al. 2020; Kamel and Iadecola 2012). In literature, hypereosinophilia has been reported as an unusual cause of AIS (Khwaja et al. 2013). A study showed that in the AIS patients who were treated with recombinant tissue plasminogen activator (rtPA) within 4.5 h of symptom onset, eosinophil count (AEC) ≥ 0.11 × 10^9^/L was independently associated with a 78% reduction in the odds of developing hemorrhagic transformation (Jucevičiūtė et al. 2019). It was also reported that patients with eosinopenia (defined as a percentage of eosinophils < 0.3%) had significantly larger infarct volumes and that AIS patients with eosinopenia at admission had significant risk factors for mortality at 2 months (Hori et al. 2016).

However, previous studies have been limited, with small sample sizes in these studies, or with end‐points that only included short‐term clinical outcomes and prognosis, or lacking larger scale multicenter prospective studies. Using the data from the Third China National Stroke Registry (CNSR‐III), we investigated the association between eosinophil counts and eosinophil ratios with clinical outcomes at a long term in patients with an ischemic stroke.

## 2 Methods

### 2.1 Study Participants

The data for this secondary analysis were derived from the CNSR‐III, a nationwide, large‐scale, multicenter prospective clinical registry study conducted across 201 hospitals in 22 provinces and four municipalities throughout China. From August 2015 to March 2018, a consecutive sampling method was used to recruit a total of 15,166 patients who had experienced an AIS or transient ischemic attack (TIA) within the preceding 7 days. Enrolled patients met the following criteria: (1) age older than 18 years; (2) diagnosed ischemic stroke or TIA, according to the WHO criteria (Stroke–1989. Recommendations on Stroke Prevention, Diagnosis, and Therapy. Report of the WHO Task Force on Stroke and other Cerebrovascular Disorders 1989) and confirmed by brain MRI or brain CT; (3) onset of symptoms to enrolment within 7 days; (4) principle of informed consent from patient or legally authorized representative. Exclusion criteria were as follows: (1) silent cerebral infarction or (2) declined registry participation. Details of the design and major results of the study have been previously published (Wang et al. 2019).

**FIGURE 1:**
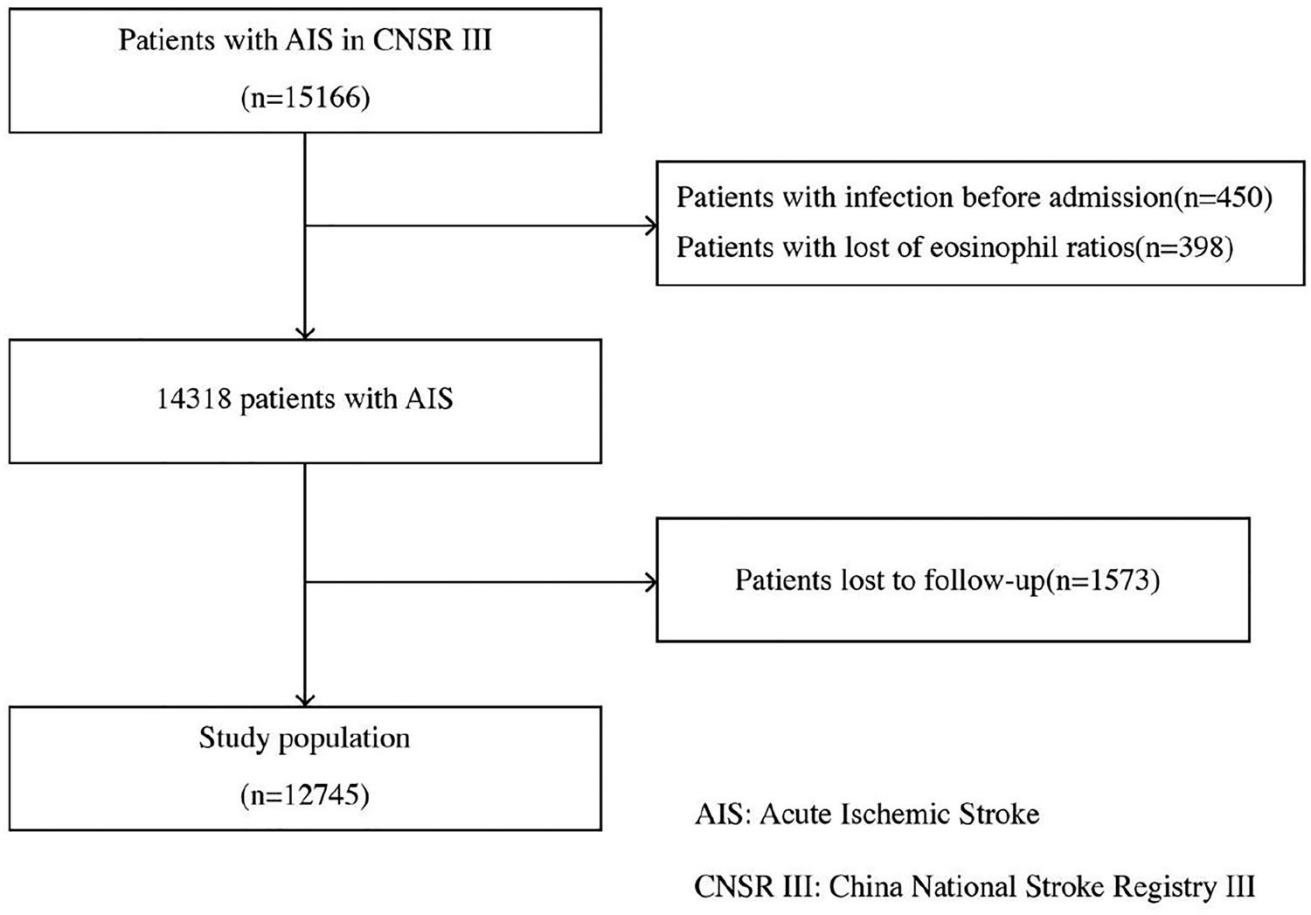
Flowchart of the study.

In this analysis, we excluded patients if they met one of the following criteria: (1) had proven infection on admission; (2) lacked available eosinophils data on admission; (3) were lost to follow‐up (Figure 1).

### 2.2 Standard Protocol Approvals, Registrations, and Patient Consents

The CNSR‐III was approved by the ethics committees of Beijing Tiantan Hospital and all other study centers (IRB approval number: KY2015‐001‐01). Written informed consent was provided to the patients or their legal representatives prior to enrollment in the study. The study was conducted following the guidelines described in the Helsinki Declaration.

### 2.3 Baseline Data Collection

Well‐trained neurologists who were from each participating hospitals collected baseline data including baseline data (age, sex, and current smoking), medical histories (stroke or TIA, hypertension, coronary artery disease, atrial fibrillation, dyslipidemia, and diabetes), and the modified Rankin Scale (mRS) score (Quinn et al. 2009) as well as the National Institutes of Health Stroke Scale (NIHSS) score (Goldstein et al. 1989) at admission by face‐to‐face interviews or through the medical records. Furthermore, intravenous thrombolysis therapy and the TOAST [Trial of Org 10172 in Acute Stroke Treatment] criteria (Adams et al. 1993) also were assessed at admission. Briefly, The TOAST classification comprises five etiological subtypes: large‐artery atherosclerosis, cardioembolism, small‐vessel occlusion, stroke of other determined etiology, and stroke of undetermined etiology.

Blood samples were collected across 171 study sites with expertise in genetic and biomarker research. At each site, fasting venous blood was drawn into EDTA tubes within 24 h of admission and kept at room temperature until processing. Following collection, eosinophil counts and ratios were analyzed at each participating hospital's laboratory according to standardized protocols, with laboratory personnel blinded to clinical outcomes.

### 2.4 Outcome Assessment

The primary efficacy outcomes were stroke recurrence, poor functional outcomes, recurrence of ischemic stroke, combined vascular events, and all‐cause death at 5 years.

Stroke recurrence, which includes ischemic and hemorrhagic stroke, was defined as a new focal neurological impairment confirmed by neuroimaging at the treating hospital. The mRS as a tool was used to assess functional status of patients. Functional outcomes were assessed using the mRS, a 7‐point ordinal scale ranging from 0 (no symptoms) to 6 (death). Consistent with conventional clinical criteria, a poor functional outcome was defined as an mRS score of 2 to 6, which encompasses all states of functional dependence (requiring assistance with daily activities) through death. Combined vascular events include cardiovascular death, nonfatal stroke, and nonfatal myocardial infarction. The definition of the fatality was either confirmed by a death certificate from the attended hospital or the local civil registry. The aforementioned clinical outcomes were observed by telephone at 5 years after the onset of the stroke. Neurological deficit and stroke severity were assessed on admission by certified neurologists using the National Institutes of Health Stroke Scale (NIHSS).

### 2.5 Statistical Analysis

Patients enrolled in the study were assigned to four groups according to the quartiles of eosinophil counts or eosinophil ratios assessed at admission. Continuous variables are expressed as means ± standard deviations (SDs) or median with interquartile range and were compared using the Kruskal–Wallis *U* test. Categorical variables are expressed as frequency with percentage and were compared using the *χ*‐test (Herpich and Rincon 2020).

The association of eosinophil counts or ratio levels with poor functional outcomes was analyzed using a logistic regression model. The Cox proportional hazard regression model was used to estimate the association of eosinophil counts or eosinophil ratios with stroke recurrence, death, ischemic stroke recurrence, and combined vascular events. Hazard ratios (HRs) or odds ratios (ORs) with 95% confidence intervals (CIs) were calculated after adjusting for potential confounding factors. Two steps were performed to adjust the covariates: In the first model, we only adjusted for age and gender. In the second model, we included a variety of potential confounding variables, including age, sex, history of hypertension, diabetes, dyslipidemia, coronary artery disease, atrial fibrillation, stroke or TIA, current smoking, admission NIHSS score, TOAST classification (including five subtypes of ischemic stroke: large‐artery atherosclerosis, cardioembolism, small‐vessel occlusion, other determined etiologies, and undetermined causes) and the number of days from symptom onset to admission. Missing data were addressed as follows: for missing baseline eosinophil values, cases were excluded from the analysis. For participants lost to follow‐up, we employed the censoring mechanism inherent in the Kaplan–Meier method at their last known contact date. A sensitivity analysis was conducted to assess the potential impact of these missing data on the primary findings.

In addition, we evaluated the pattern of association between eosinophil counts or eosinophil ratios on a continuous scale and the risk of adverse clinical outcomes at 5‐year follow‐up using a Cox regression model with restricted cubic splines for eosinophil counts or ratios adjusted for potential covariates. Significance is defined as a *p* value less than 0.05. All statistical analyses were performed using SAS software version 9.4 (SAS Institute Inc).

## 3 Results

### 3.1 Study Participants and Characteristics

A total of 15,166 patients diagnosed minor ischemic stroke or TIA were enrolled in the CNSR‐III. After excluding 450 patients with infection (including respiratory and urinary infection) before admission, 398 patients without an available eosinophil ratios on admission and 1573 patients lost to follow‐up; Figure 1 shows that a total of 12,745 patients were included in our analysis. The baseline characteristics of the participant patients were classified into four groups based on eosinophil counts and eosinophil ratios, as shown in Tables 1 and 2.

**TABLE 1:** Baseline characteristics of the patients by eosinophils count quartiles.

Variables		Eosinophils count, ×10^9^/L				*p* value
	Overall	Q4, ≥ 0.17	Q3, 0.10–0.16	Q2, 0.06–0.09	Q1, ≤ 0.05	
	*n* = 12,513	*n* = 3317	*n* = 3225	*n* = 3143	*n* = 2828	
Age, median (IQR), years	62 (54–70)	62 (54–69)	62 (54–70)	63 (54–70)	63 (55–70)	< 0.001
Female, *n* (%)	4015 (32.09)	713 (21.50)	985 (30.54)	1141 (36.30)	1176 (41.58)	<0.0001
History, *n* (%)						
Hypertension	7833 (62.60)	2061 (62.13)	2013 (62.42)	1985 (63.16)	1774 (62.73)	0.85
Diabetes	2926 (23.38)	790 (23.82)	800 (24.81)	756 (24.05)	580 (20.51)	<0.001
Dyslipidemia	996 (7.96)	299 (9.01)	280 (8.68)	243 (7.73)	174 (6.15)	<0.001
Coronary artery disease	1317 (10.53)	319 (9.62)	321 (9.95)	376 (11.96)	301 (10.64)	0.01
Atrial fibrillation	820 (6.55)	166 (5.00)	180 (5.58)	214 (6.81)	260 (9.19)	<0.0001
Stroke or TIA	3025 (24.17)	826 (24.90)	772 (23.94)	758 (24.12)	669 (23.66)	0.69
Current smoking	3891 (31.10)	1307 (39.40)	1076 (33.36)	867 (27.59)	641 (22.67)	<0.0001
Admission NIHSS score, median (IQR)	3 (1–5)	3 (1–5)	3 (1–5)	3 (1–5)	4 (2–7)	<0.0001
TOAST, *n* (%)						<0.0001
Large‐artery atherosclerosis	3129 (25.01)	817 (24.63)	719 (22.29)	801 (25.49)	792 (28.01)	
Cardioembolism	737 (5.89)	168 (5.06)	187 (5.80)	194 (6.17)	188 (6.65)	
Small‐vessel occlusion	2686 (21.47)	740 (22.31)	717 (22.23)	710 (22.59)	519 (18.35)	
Other determined etiology	152 (1.21)	44 (1.33)	33 (1.02)	35 (1.11)	40 (1.41)	
Undetermined etiology	5809 (46.42)	1548 (46.67)	1569 (48.65)	1403 (44.64)	1289 (45.58)	
Days from onset of symptoms to admission, mean (SD)	1.09 ± 1.51	1.14 ± 1.54	1.16 ± 1.50	1.10 ± 1.60	0.95 ± 1.35	<0.0001

*Note*: Continuous variables are expressed as median with interquartile range. Categorical variables are expressed as frequency with percentage.

Abbreviations: IQR, interquartile range; NIHSS, The National Institutes of Health Stroke Scale; TIA, transient ischemic Attack; TOAST, Trial of Org 10,172 in Acute Stroke Treatment.

**TABLE 2:** Baseline characteristics of the patients by eosinophils ratio quartiles.

Variables		Eosinophils ratio, %				*p* value
	Overall	Q4, ≥ 2.50	Q3, 1.46–2.49	Q2, 0.74–1.45	Q1, ≤ 0.73	
	*n* = 12,513	*n* = 3128	*n* = 3130	*n* = 3124	*n* = 3131	
Age, median (IQR), years	62 (54–70)	62 (54–70)	62 (54–69)	62 (54–70)	63 (54–70)	0.02
Female, *n* (%)	4015 (32.09)	725 (23.18)	943 (30.13)	1084 (34.70)	1263 (40.34)	<0.0001
History, *n* (%)						
Hypertension	7833 (62.60)	1932 (61.76)	1930 (61.66)	1995 (63.86)	1976 (63.11)	0.21
Diabetes	2926 (23.38)	698 (22.31)	779 (24.89)	789 (25.26)	660 (21.08)	<0.0001
Dyslipidemia	996 (7.96)	266 (8.50)	277 (8.85)	259 (8.29)	194 (6.20)	<0.001
Coronary artery disease	1317 (10.53)	309 (9.88)	323 (10.32)	346 (11.08)	339 (10.83)	0.42
Atrial fibrillation	820 (6.55)	163 (5.21)	168 (5.37)	197 (6.31)	292 (9.33)	<0.0001
Stroke or TIA	3025 (24.17)	788 (25.19)	732 (23.39)	784 (25.10)	721 (23.03)	0.09
Current smoking	3891 (31.10)	1167 (37.31)	1038 (33.16)	922 (29.51)	764 (24.40)	<0.0001
Admission NIHSS score, median (IQR)	3 (1‐6)	3 (1‐5)	3 (1‐5)	3 (1‐5)	4 (2‐7)	<0.0001
TOAST, *n* (%)						<0.0001
Large‐artery atherosclerosis	3129 (25.01)	732 (23.40)	709 (22.65)	776 (24.84)	912 (29.13)	
Cardioembolism	737 (5.89)	167 (5.34)	173 (5.53)	189 (6.05)	208 (6.64)	
Small‐vessel occlusion	2686 (21.47)	736 (23.53)	719 (22.97)	679 (21.73)	552 (17.63)	
Other determined etiology	152 (1.21)	41 (1.31)	32 (1.02)	38 (1.22)	41 (1.31)	
Undetermined etiology	5809 (46.42)	1452 (46.42)	1497 (47.83)	1442 (46.16)	1418 (45.29)	
Days from onset of symptoms to admission, mean (SD)	1.09 ± 1.51	1.17 ± 1.55	1.18 ± 1.51	1.07 ± 1.59	0.95 ± 1.37	<0.0001

*Note*: Continuous variables are expressed as median with interquartile range. Categorical variables are expressed as frequency with percentage.

Abbreviations: IQR, interquartile range; NIHSS, The National Institutes of Health Stroke Scale; TIA, transient ischemic attack; TOAST, Trial of Org 10,172 in Acute Stroke Treatment.

Compared with patients in the higher eosinophil counts quartile, those in the lower quartile were more likely to be female and older, had a higher median admission NIHSS score, were more frequently classified as the large vessel atherosclerosis or cardioembolic subtype (according to The Trial of Org 10172 in Acute Stroke Treatment (TOAST) criteria), and had a history of atrial fibrillation at admission. Moreover, patients in the lower eosinophil count group were less likely to be current smokers or to have a history of dyslipidemia, coronary artery disease, or diabetes. Additionally, the time from symptom onset to admission was shorter in this group (Table 1). Participants in the lower eosinophil ratios quartile group were in the same situation as participants in the lower eosinophil counts quartile group generally, but patients in lower eosinophil ratios quartile group did not have statistical significance of coronary artery disease (Table 2).

### 3.2 Association of Eosinophils Counts and Ratio With Clinical Outcomes

During 5‐year follow‐up, 1992 (15.92%) patients had stroke recurrence, of whom 1833 (14.65%) were ischemic stroke, 3626 (28.98%) had poor functional outcome whose mRS score between 2 and 6, 1386 (11.08%) had all‐cause death, and 2302 (18.40%) had new combined vascular events.

Table 3 shows the association between quartile eosinophil counts and clinical outcomes. Patients in the lower eosinophil categories were significantly associated with clinical outcomes deterioration at 5 years. In the first model, after adjusting for potential confounders like age and gender, patients with the eosinophil counts in quartile 1 had a higher risk of stroke recurrence, which showed a 25% (adjusted HR, 1.25; 95% CI: 1.10–1.41) increase than the patients in higher quartiles of eosinophil counts at 5 years. After adjusting for multiple potential confounders, as the model 2 showed, the association also persisted (adjusted HR, 1.19; 95% CI: 1.05–1.35). Regarding the ischemic stroke recurrence, patients with eosinophil counts in quartile 1 also had increased risk of adverse outcomes after 5 years (adjusted HR, 1.25; 95% CI: 1.10–1.42). This association persisted after adjustment for potential confounders. Moreover, similar results were observed for poor functional outcomes which mRS score between 2 and 6, all‐cause death, and combined vascular events. After a period of 5 years follow‐up, the incidence of poor functional outcomes (mRS 2–6), all‐cause death, and combined vascular events in patients with lowest quartile was increased, which revealed adjusted odds ratio of 1.57 (95% CI: 1.40–1.76) and adjusted hazard ratio of 1.53 (95% CI: 1.32–1.77) and 1.15(95% CI: 1.02–1.29), respectively. Model 2 also shows the same increasing trend, which is also statistically significant.

**TABLE 3:** Association of clinical outcomes with quartile of eosinophil count.

Outcomes	Quartile of eosinophils count, ×10^9^/L	No.	Events, *n* (%)	Model 1^a^		Model 2^b^	
				Adjusted HR/OR (95% CI)	*p* value	Adjusted HR/OR (95% CI)^c^	*p* value
**At 5 years**							
Stroke recurrence	Q4, ≥ 0.17	3317	507 (15.28)	1.00 (Ref)		1.00 (Ref)	
	Q3, 0.10–0.16	3225	462 (14.33)	0.93 (0.82–1.06)	0.29	0.94 (0.83–1.07)	0.34
	Q2, 0.06–0.09	3143	498 (15.84)	1.03 (0.91–1.16)	0.66	1.03 (0.91–1.17)	0.64
	Q1, ≤ 0.05	2828	525 (18.56)	1.25 (1.10–1.41)	<0.001	1.19 (1.05–1.35)	0.006
Ischemic stroke	Q4, ≥ 0.17	3317	463 (13.96)	1.00 (Ref)		1.00 (Ref)	
	Q3, 0.10–0.16	3225	428 (13.27)	0.95 (0.83–1.08)	0.40	0.95 (0.83–1.08)	0.42
	Q2, 0.06–0.09	3143	458 (14.57)	1.03 (0.90–1.17)	0.67	1.03 (0.90–1.17)	0.70
	Q1, ≤ 0.05	2828	484 (17.11)	1.25 (1.10–1.42)	<0.001	1.20 (1.05–1.37)	0.007
mRS 2–6	Q4, ≥ 0.17	3317	870(26.23)	1.00 (Ref)		1.00 (Ref)	
	Q3, 0.10–0.16	3225	881(27.32)	1.05 (0.93–1.18)	0.42	1.07 (0.95–1.21)	0.27
	Q2, 0.06–0.09	3143	850(27.04)	1.01 (0.90–1.14)	0.82	1.01 (0.90–1.14)	0.83
	Q1, ≤ 0.05	2828	1025(36.24)	1.57 (1.40–1.76)	<0.0001	1.35 (1.20–1.53)	<0.0001
Combined vascular events^d^	Q4, ≥ 0.17	3317	613 (18.48)	1.00 (Ref)		1.00 (Ref)	
	Q3, 0.10–0.16	3225	539 (16.71)	0.90 (0.80–1.01)	0.06	0.90 (0.80–1.01)	0.08
	Q2, 0.06–0.09	3143	564 (17.94)	0.96 (0.86–1.08)	0.49	0.96 (0.86–1.08)	0.50
	Q1, ≤ 0.05	2828	586 (20.72)	1.15 (1.02–1.29)	0.02	1.11 (0.99–1.24)	0.09
All‐cause death	Q4, ≥ 0.17	3317	330 (9.95)	1.00 (Ref)		1.00 (Ref)	
	Q3, 0.10–0.16	3225	331 (10.26)	1.03 (0.89–1.20)	0.69	1.05 (0.90–1.22)	0.56
	Q2, 0.06–0.09	3143	300 (9.55)	0.94 (0.81–1.10)	0.46	0.94 (0.80–1.10)	0.45
	Q1, ≤ 0.05	2828	425 (15.03)	1.53 (1.32–1.77)	<0.0001	1.29 (1.11–1.49)	0.001

Abbreviations: CI, confidence interval; HR, hazard ratio; mRS modified Rankin Scale; OR, odds ratio; Ref, reference.

^a^ Model 1: Adjusted for age and gender.

^b^ Model 2: Adjusted for age, gender, history of ischemic stroke, TIA, myocardial infarction, angina, congestive heart failure, known atrial fibrillation or flutter, valvular heart disease, hypertension, diabetes mellitus, hypercholesterolemia, smoking status, index event and NIH stroke scale on admission, time to randomization, and antiplatelet therapy.

^c^ Hazard ratios (HRs) with 95% confidence intervals (CIs) were used for stroke recurrence, ischemic stroke, all‐cause death, and combined vascular events; odds ratios (ORs) with 95% CIs were used for mRS 2–6.

^d^ Combined vascular event: Occurrence of combined vascular event including cardiovascular death, non‐fatal stroke, and non‐fatal myocardial infarction.

**FIGURE 2:**
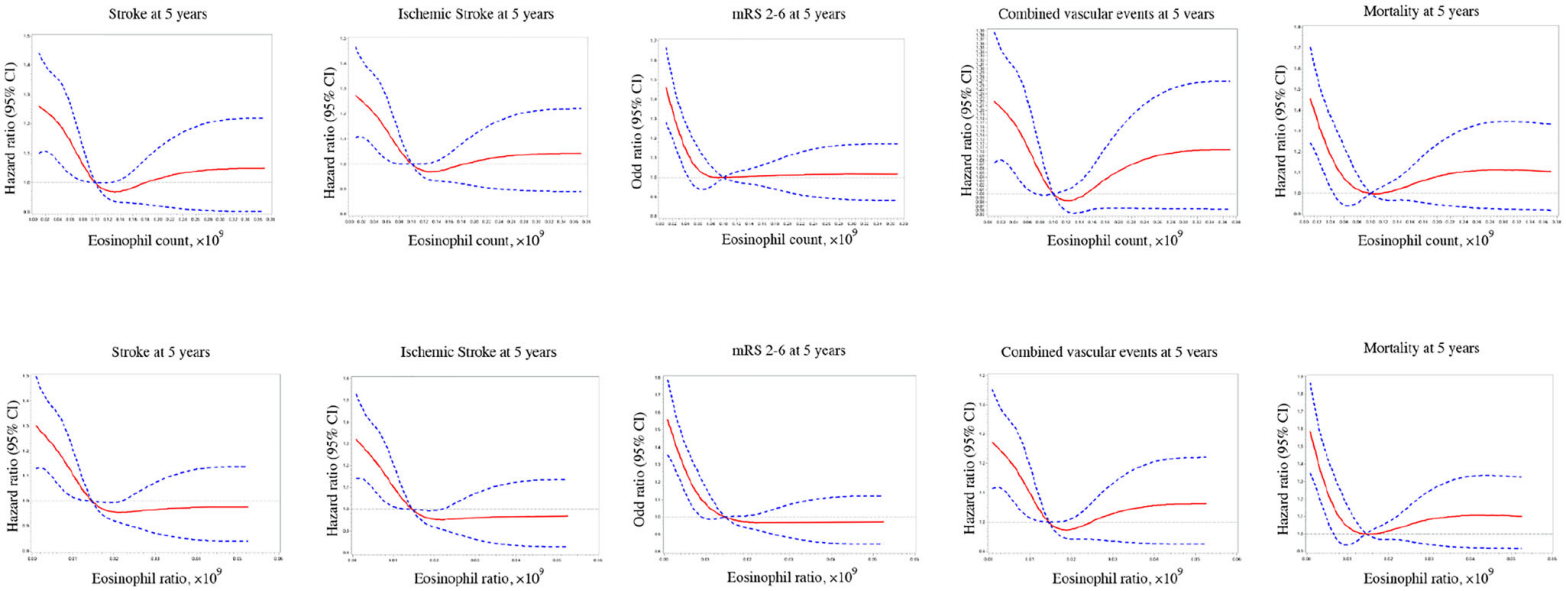
Associations of adverse clinical outcomes at 5‐year follow‐up with eosinophils counts and eosinophils ratio. Red lines indicate adjusted HRs or ORs. Blue lines indicate 95% CI bands. Data were fitted with the multivariable Cox regression model of restricted cubic spline for eosinophils count and eosinophils ratio, with adjustment for potential covariates. mRS, modified Rankin Scale; HR, hazard ratio; OR, odds ratio; CI, confidence interval.

As shown in Table 4, we found similar association using the eosinophil ratios instead of the eosinophil counts. Patients in the lower eosinophil ratios category were associated with worse clinical outcomes at 5 years, including a higher risk of recurrence of stroke, new ischemic stroke, poor functional outcomes, all‐cause death, and composite vascular events at 5 years. After adjusting confounding variables like age and gender, lowest eosinophil ratios were, respectively, associated with increased risk of stroke recurrence (adjusted HR, 1.36; 95% CI: 1.20–1.54) and ischemic stroke recurrence (adjusted HR, 1.37; 95% CI: 1.21–1.57). The above relation was still significant after adjustment in Model 2. As for the remaining clinical outcomes, there was also a significant correlation. Patients with the eosinophil ratios level in quartile 1 had risks of adverse clinical outcomes such as poor functional outcomes which the mRS score between 2 and 6 (adjusted OR, 1.74; 95% CI: 1.55–1.95), all‐cause death (adjusted HR, 1.67; 95% CI: 1.45–1.93), and combined vascular events (adjusted HR, 1.24; 95% CI: 1.10–1.39). In addition, the same increasing trend was found to be statistically significant after adjustment for multiple latent factors. Multivariable‐adjusted spline regression models of the association between eosinophil counts and eosinophil ratios and adverse clinical outcomes, including stroke recurrence, poor functional outcomes, combined vascular events, and all‐cause death at 5‐year follow‐up are shown in Figure 2.

**TABLE 4:** Association of clinical outcomes with quartile of eosinophil ratio.

Outcomes	Quartile of eosinophils ratio, ×10^9^/L	No.	Events, *n* (%)	Model 1^a^		Model 2^b^	
				Adjusted HR/OR (95% CI)	*p* value	Adjusted HR/OR (95% CI)^c^	*p* value
**At 5 years**							
Stroke recurrence	Q4, ≥ 2.50	3128	456 (14.58)	1.00 (Ref)		1.00 (Ref)	
	Q3, 1.46–2.49	3130	445 (14.22)	0.99 (0.87–1.13)	0.90	0.99 (0.87–1.13)	0.87
	Q2, 0.74–1.45	3124	504 (16.13)	1.13 (0.99–1.28)	0.07	1.11 (0.98–1.26)	0.12
	Q1, ≤ 0.73	3131	587 (18.75)	1.36 (1.20–1.54)	<0.0001	1.29 (1.13–1.46)	<0.0001
Ischemic stroke	Q4, ≥ 2.50	3128	416 (13.30)	1.00 (Ref)		1.00 (Ref)	
	Q3, 1.46–2.49	3130	406 (12.97)	0.99 (0.86–1.13)	0.86	0.98 (0.86–1.13)	0.79
	Q2, 0.74–1.45	3124	467 (14.95)	1.14 (1.00–1.30)	0.05	1.12 (0.98–1.27)	0.11
	Q1, ≤ 0.73	3131	544 (17.37)	1.37 (1.21–1.57)	<0.0001	1.30 (1.14–1.49)	<0.0001
mRS 2–6	Q4, ≥ 2.50	3128	819 (26.18)	1.00 (Ref)		1.00 (Ref)	
	Q3, 1.46–2.49	3130	804 (25.69)	1.03 (0.92–1.16)	0.59	1.01 (0.89–1.14)	0.90
	Q2, 0.74–1.45	3124	853 (27.30)	1.11 (0.98–1.24)	0.09	1.07 (0.94–1.20)	0.30
	Q1, ≤ 0.73	3131	1150 (36.73)	1.74 (1.55–1.95)	<0.0001	1.44 (1.28–1.63)	<0.0001
Combined vascular events^d^	Q4, ≥ 2.50	3128	558 (17.84)	1.00 (Ref)		1.00 (Ref)	
	Q3, 1.46–2.49	3130	517 (16.52)	0.94(0.83–1.06)	0.29	0.94 (0.83–1.06)	0.27
	Q2, 0.74–1.45	3124	574 (18.37)	1.05 (0.93–1.18)	0.45	1.03 (0.92–1.16)	0.63
	Q1, ≤ 0.73	3131	653 (20.86)	1.24 (1.10–1.39)	<0.001	1.18 (1.05–1.32)	0.006
All‐cause death	Q4, ≥ 2.50	3128	318 (10.17)	1.00 (Ref)		1.00 (Ref)	
	Q3, 1.46–2.49	3130	307 (9.81)	1.06 (0.91–1.24)	0.46	1.03 (0.88–1.20)	0.74
	Q2, 0.74–1.45	3124	282 (9.03)	0.94 (0.80–1.10)	0.43	0.90 (0.77–1.06)	0.20
	Q1, ≤ 0.73	3131	479 (15.30)	1.67 (1.45–1.93)	<.0001	1.36 (1.17–1.57)	<0.0001

Abbreviations: CI, confidence interval; HR, hazard ratio; mRS, modified Rankin Scale; OR, odds ratio; Ref, reference.

^a^ Model 1: Adjusted for age and gender.

^b^ Model 2: Adjusted for age, gender, history of ischemic stroke, TIA, myocardial infarction, angina, congestive heart failure, known atrial fibrillation or flutter, valvular heart disease, hypertension, diabetes mellitus, hypercholesterolemia, smoking status, index event and NIH stroke scale on admission, time to randomization, and antiplatelet therapy.

^c^ Hazard ratios (HRs) with 95% confidence intervals (CIs) were used for stroke recurrence, ischemic stroke, all‐cause death, and combined vascular events; odds ratios (ORs) with 95% CIs were used for mRS 2–6.

^d^ Combined vascular event: Occurrence of combined vascular event including cardiovascular death, non‐fatal stroke, and non‐fatal myocardial infarction.

**FIGURE 3:**
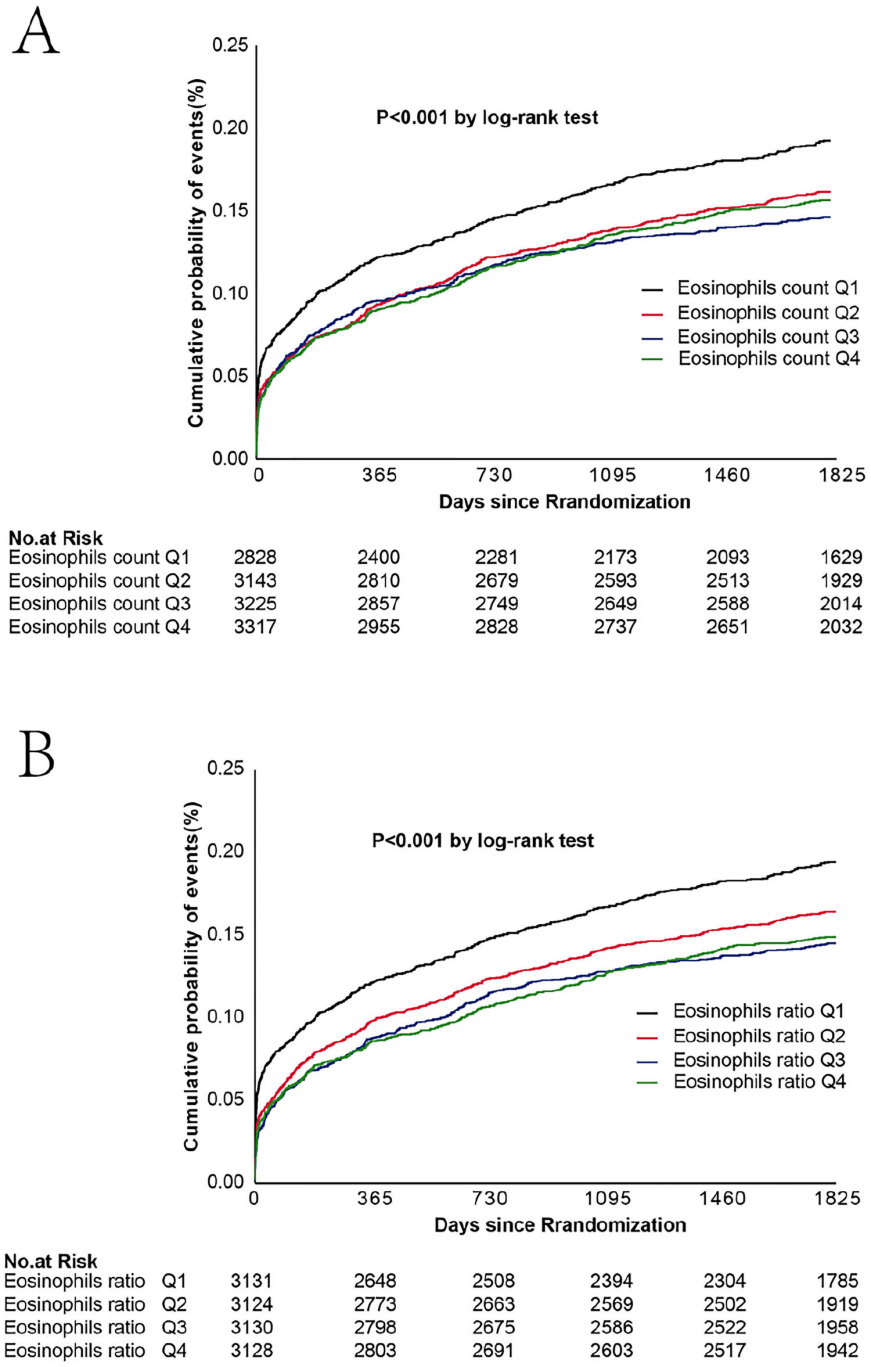
K–M survival analysis curves for eosinophils counts (A) and eosinophils ratio (B) and incidence of cumulative probability of events at 5 years.

To evaluate the 5‐year cumulative incidence of events based on eosinophil counts and ratios, we employed Kaplan–Meier (K–M) curves (Figure 3). In panel A, patients were stratified by their absolute eosinophil count. The results demonstrated a significant survival advantage for the group with high eosinophil counts, as their event‐free survival probability was consistently higher over the 5‐year follow‐up period compared to the low‐count group. Panel B presents a similar analysis based on the eosinophil ratio, the findings of which were consistent with those in panel A. Collectively, the K–M analysis demonstrated that patients with the lowest eosinophil counts and ratios faced an increased long‐term risk of adverse clinical outcomes, as evidenced by a significantly higher cumulative event probability (log‐rank *p* < 0.001).

## 4 Discussion

In the CNSR‐III, which is the large prospective multicenter cohort analysis, we found that both lowest level of eosinophils counts and eosinophils ratio were associated with increased risk of adverse clinical outcomes in patients with a minor ischemic stroke or TIA at 5 years.

Cerebral artery thrombosis, also known as ischemic stroke, is the most common form of arterial thrombosis that affects the health of patients (Xu et al. 2022). AIS is no longer considered a vascular disease, and inflammation has been identified as a contributor to the pathophysiology of AIS. Neuroinflammation affects the outcome of both ischemic reperfusion injury and permanent ischemic disease (Stoll and Nieswandt 2019). Eosinophils, as pleiotropic multifunctional leukocytes, participate in the initiation and propagation of multiple inflammatory responses, as well as innate and adaptive immunoregulation (Hogan et al. 2008). The reduction of eosinophils can be caused by acute inflammation through mechanisms such as diffusing margination of eosinophils or sequestration of the eosinophil within an organ (Bass et al. 1980). In addition to constituting only 1%–8% of peripheral blood leukocytes, eosinophils play an important role in the vascular innate immune system by directly contributing to the coagulation and fibrinolysis systems through the production of key factors such as tissue factor, thrombin, and plasminogen (Coden and Berdnikovs 2020). Eosinophils can produce, store, and release a broad spectrum of biologically active substances—including chemokines, cytotoxic proteins, lipid mediators, and over 35 distinct inflammatory and regulatory cytokines—that mediate and regulate inflammation (Hogan et al. 2008; Gleich 2000). However, the exact mechanisms underlying the relationship between eosinopenia and adverse clinical outcomes in patients with AIS remain unclear. On the one hand, nerve growth factor (NGF) and vascular endothelial growth factor (VEGF) can be produced by eosinophils that may play an important role in the functional recovery after AIS (Puxeddu et al. 2005). On the other hand, eosinophil can also lead to reactive oxygen species (ROS) and cysteinyl leukotrienes generation by shaping change, which contributes to leukocyte infiltration blood–brain barrier (BBB) (Bolton et al. 2003). Another study found that cytotoxic protein‐mediated thrombosis could be caused by the degranulation of eosinophils and endothelial injury to promote brain infarction (Wang et al. 2017).

Recently, there were increased interests in the using of eosinophil counts and ratios as predictors of cerebral or coronary thrombosis disease. However, results on the clinical prognosis of eosinophil counts or ratios in coronary or cerebral thrombosis disordered vary widely across studies. In the analysis of included studies of cardiovascular events, increased eosinophil counts were strongly related to increased risks of both non‐ST‐elevation myocardial infarction and ST‐elevation myocardial infarction in the coronary artery disease population, with an OR of 1.37 (95% CI: 1.26–1.49, *p* < 0.00001) (Gao et al. 2019). Eosinophils had also been linked to cardiac tissue repair and subsequent adverse remodeling (Han et al. 2022; Rios‐Navarro et al. 2018). A few reports had studied the severity of stroke in relation to eosinophils. A pilot study showed that among 3‐month clinical outcomes, patients with higher AEC (eosinophil counts) were better than those who had normal or lower eosinophil counts (Guo et al. 2015). However, this literature only admitted the AIS patients (“acute” was defined as those patients who were diagnosed within 24 h) who received a standard thrombolytic therapy and did not exclude patients who had infection before admission. Recent studies also showed a strong association between eosinopenia and mortality and high infection rates in ischemic stroke patients. In this analysis, eosinopenia was found to be associated with a poor short‐term prognosis. Ischemic stroke patients with eosinopenia had higher mortality, increased prevalence of infection, and higher prevalence of death associated with infection (Hori et al. 2016). Still, the study had limitations in that the endpoint included only short‐term mortality and lacked a long‐term endpoint. As for eosinophil percentage, a study showed that compared with other three higher quartiles, patients with the lowest eosinophil percentage quartile (< 0.4%) had a higher rate of infection, larger infarct volume, and poorer clinical outcomes (Zhao et al. 2019). However, the study did not explore the correlation between eosinophil counts and clinical outcomes and lacked a long‐term endpoint. Our results are consistent with previous studies, demonstrating the prognostic significance of eosinopenia and clinical outcomes in patients with AIS. This study also emphasizes the importance of exploring the deep relationship between eosinophils and clinical functional prognosis.

The main strength of our program is that this is an analysis of a multicenter, prospective cohort study with a large number of cases to date and with long‐term follow‐up. In addition, we adjusted for as many confounding factors as available, such as age, sex, and history of diabetes. Elevated eosinophil counts or ratios represent readily available prognostic parameters. The determination of eosinophil counts and eosinophil ratios is economical, rapid, and universally available. Our study provides an easily measurable marker that has been shown to be clinically relevant over long follow‐up, which may improve our ability to stratify the risk of minor ischemic stroke or TIA in clinical practice. However, there are some limitations to this study that should be noted. First, it was a secondary data analysis, with blood samples collected at different times that may have affected eosinophil counts and ratios. Second, the exclusion criteria did not contain the people with histories of asthma, parasitic infections, fungal infections, viral infections, and hypereosinophilic syndrome (Klion et al. 2020), which were associated with the decrease or increase in circulating eosinophils in the blood. Third, our analysis relied on a single baseline measurement of eosinophils. Although this practical approach proved highly informative for long‐term risk prediction, it cannot capture potential fluctuations in the post‐acute phase. Finally, thrombus samples from the infarct region of AIS patients could not be obtained directly, leading us to no direct evidence that eosinophil promotes thrombosis formation and growth. Hence, we may need to pay more attentions to these limitations in further research design and recruitment. Inflammatory parameters such as eosinophil counts and eosinophil ratios were associated with an increased risk of adverse clinical outcomes, all‐cause death, composite events, and ischemic stroke in patients with minor ischemic stroke or TIA.

Overall, this literature reveals that patients in the lowest eosinophil quartile had a significantly increased risk of major adverse clinical outcomes, including stroke recurrence, death, and poor functional recovery. Eosinophils are transitioning from an overlooked component of routine blood tests to a promising prognostic biomarker in AIS, with a clear developmental pathway envisioned. The short‐term (1–3 years) focus lies on validating their prognostic value across diverse populations and developing integrated multivariable models for clinical risk stratification. In the mid‐term (3–5 years), research is expected to elucidate the precise pathophysiological mechanisms of eosinophils in stroke recovery and explore their utility in monitoring post‐stroke immune status. Looking further ahead (5+ years), if supported by mechanistic evidence, the initiation of proof‐of‐concept clinical trials testing eosinophil‐targeted immunomodulatory therapies could open a novel avenue for stroke treatment. In summary, their potential as an inexpensive, accessible biomarker and a component of a modifiable immunomodulatory pathway positions eosinophils to play a significant role in the future of precision medicine for ischemic stroke.

Based on long‐term follow‐up data from a large‐scale prospective cohort, this study demonstrates for the first time that low levels of peripheral blood eosinophil counts and ratios are independent and robust predictors of 5‐year adverse clinical outcomes in patients with ischemic stroke or TIA, showing a clear dose–response relationship. The findings underscore the significant value of eosinophils as a prognostic biomarker for long‐term stroke outcomes, not only providing a novel basis for risk stratification but also laying crucial groundwork for exploring their role in post‐stroke long‐term pathological mechanisms and identifying potential therapeutic targets.

## Author Contributions

Study concept and design, analysis and interpretation of data, and drafting of the manuscript: Yangying Lu. Acquisition of data and drafting of the manuscript: Lei Zhang. Acquisition of data: Yuesong Pan, Hongyi Yan, Xia Meng, Jing Jing, Honglian Wang, and Wenchao Li. Acquisition of data, study supervision, or coordination: Xingquan Zhao, Liping Liu, and Yilong Wang. Acquisition of data, study supervision, or coordination: Yongjun Wang. Study concept and design, acquisition of data, analysis, and interpretation of data: Bihong Zhu.

## Funding

This study is supported by grants from Zhejiang Municipal Science and Technology Commission (LTGY23H090003).

## Conflicts of Interest

The authors declare no conflicts of interest.

## Data Availability

The data that support the findings of this study are not openly available due to reasons of sensitivity and are available from the corresponding author upon reasonable request.
